# Involvement of Rab6a in organelle rearrangement and cytoskeletal organization during mouse oocyte maturation

**DOI:** 10.1038/srep23560

**Published:** 2016-03-31

**Authors:** Rujun Ma, Jiaqi Zhang, Xiaohui Liu, Ling Li, Honglin Liu, Rong Rui, Ling Gu, Qiang Wang

**Affiliations:** 1 College of Animal Science & Technology, Nanjing Agricultural University, Nanjing, China; 2 State Key Laboratory of Reproductive Medicine, Nanjing Medical University, Nanjing, China; 3 Center of Reproductive Medicine, Jinling Hospital, Medical School of Nanjing University, Nanjing, China; 4 College of Veterinary Medicine, Nanjing Agricultural University, Nanjing, China

## Abstract

Rab GTPases have been reported to define the identity and transport routes of vesicles. Rab6 is one of the most extensively studied Rab proteins involved in regulating organelle trafficking and integrity maintenance. However, to date, the function of Rab6 in mammalian oocytes has not been addressed. Here we report severe disorganization of endoplasmic reticulum upon specific knockdown of Rab6a in mouse oocytes. In line with this finding, intracellular Ca^2+^ stores are accordingly reduced in Rab6a-depleted oocytes. Furthermore, in these oocytes, we observe the absence of cortical granule free domain, which is a kind of special organelle in matured oocytes and its exocytosis is calcium dependent. On the other hand, following Rab6a knockdown, the prominent defects of cytoskeletal structures are detected during oocyte meiosis. In particular, the majority of Rab6a-depleted oocytes fail to form the actin cap, and the frequency of spindle defects and chromosome misalignment is significantly elevated. In summary, our data reveal that Rab6a not only participates in modulating the organization of oocyte organelles, but also is a novel regulator of meiotic apparatus in mammalian oocytes.

To produce fertilizable haploid gametes, mammalian oocytes must undergo well regulated meiotic maturation of both nucleus and cytoplasm, including resumption of meiosis, proper spindle assembly and polar body extrusion. The rearrangement of organelles, microtubules, actin filaments, and other cytoskeleton-associated proteins provides the framework for these dynamic processes^1^. Accurate control of spindle assembly and chromosome movement is required for orderly meiosis in oocytes. Any error in this process can lead to the generation of aneuploid eggs, which in humans is a major cause of pregnancy loss and developmental disabilities^2^. Although numerous molecules have been reported to be involved during oocyte maturation, pathways and mechanisms that modulate this process remain to be discovered.

Rab proteins are small GTPases that are the central regulatory factors of vesicular traffic. So far, over 60 members have been found in humans^3^. By binding to the effector molecules, Rab proteins regulate membrane trafficking, including fission, tether and fusion of intracellular vesicles through constant cycling between a membrane associated GTP-bound active and cytosolic GDP-bound inactive state^4^. Besides the membrane trafficking events, Rab proteins also regulate the vesicle transport along the actin or microtubule cytoskeleton^5^. It has been reported that Rab27a regulates melanosome transport to the plasma membrane in melanocytes via effector melanophilin^6^,^7^. Our previous work showed that Rab5a is essential for spindle length control and kinetochore-microtubule attachment during meiosis in mouse oocytes^8^. Moreover, Holubcova *et al*., revealed that vesicles positive for Rab11a modulate an actin network for asymmetric spindle positioning in oocytes^9^.

Rab6 is one of the few Rab proteins known to be involved in direct regulation of membrane transport^10^,^11^. Four different isoforms of Rab6 proteins have been identified in mammalian cells, including Rab6a, Rab6a’, Rab6b and Rab6c^12^. Rab6a and Rab6a’ are the products of alternate splicing of Rab6a gene^13^. Rab6b shows 91% identity to Rab6a and is predominantly expressed in brain^14^. Rab6c, which shows 75% identity to Rab6a’, is only expressed in brain, breast, testis and prostate^15^. Of note, Rab6a was demonstrated to regulate the dynamics of the dynein/dynactin complex at the kinetochores and consequently inactivating the spindle assembly checkpoint (SAC)^16^. Recent findings have suggested that Rab6a participates in cortical granules movement during oocyte to embryo transition in *C. elegans*^17^.

To date, however, the potential function of Rab6 in mammalian oocytes has not been addressed yet. In the present study, we set out to investigate the role of Rab6a during mouse oocyte maturation by employing siRNA knockdown. Our data suggested that Rab6a is involved in organelle rearrangement and cytoskeletal organization in oocytes.

## Results

### Cellular distribution of Rab6a during oocyte maturation

**Figure 1:**
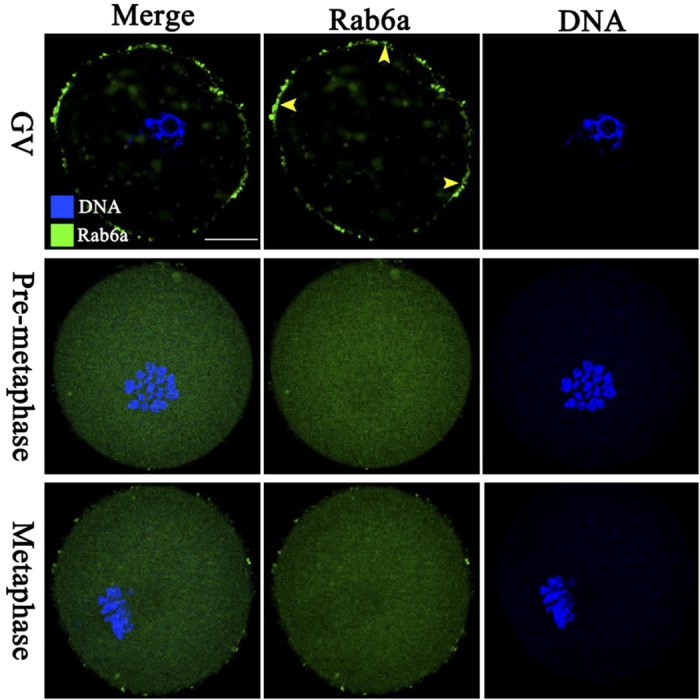
Cellular localization of Rab6a in mouse oocytes. Oocytes at GV, Pre-Metaphase I and Metaphase II stages were immunostained with Rab6a antibody (green) and counterstained with Hoechst 33342 for DNA (blue). Representative confocal images are shown. Rab6a signals on oocyte cortex are indicated by arrowheads. 30 oocytes were examined for each group. Scale bar, 25 μm.

In the current study, we first examined Rab6a distribution during mouse oocyte maturation. Immunostaining showed the presence of Rab6a in oocytes at different developmental stages (Fig. 1). These fluorescence signals predominantly reside in the membrane of fully-grown immature oocytes (arrowheads), and some of them appear to be clustered in the cytoplasm. As the oocytes enter pre-metaphase stage (GVBD +3 hours), Rab6a signals spread evenly in the whole cytoplasm. Similar distribution pattern was also observed in metaphase oocytes. Such a dynamic distribution pattern indicates that Rab6a may be involved in regulation of meiotic maturation.

### Rab6a knockdown affects meiotic progression of oocytes

**Figure 2:**
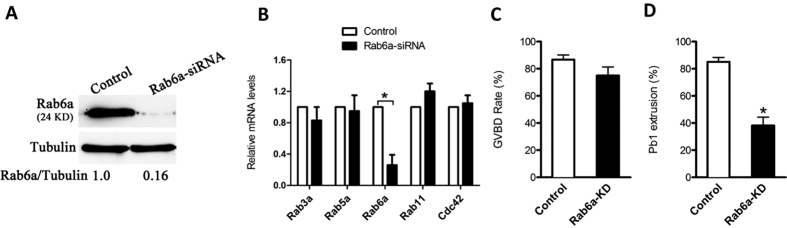
Effects of Rab6a knockdown on oocyte maturation. Fully-grown oocytes injected with Rab6a-siRNA were arrested at GV stage with milrinone for 20 hours, and then cultured in milrinone-free medium to evaluate the maturational progression. Negative control siRNA was injected as control. (**A**) Western blot showing partial knockdown of Rab6a after siRNA injection, with tubulin as a loading control. Band intensity was calculated by ImageJ software, and the ratio of Rab6a/tubulin expression was normalized and values are indicated. (**B**) The relative mRNA levels of several GTPases were determined by qRT-PCR in control and Rab6a-siRNA injected oocytes. mRNA levels in control oocytes were set as 1. (**C,D**) The rate of GVBD and Pb1 extrusion in control (n = 150) and Rab6a-KD (n = 120) oocytes. Data are expressed as mean ± SD from three independent experiments. *p < 0.05 vs control.

Next, to explore the function of Rab6a in oocyte meiosis, we microinjected the Rab6a-siRNA into fully-grown oocytes. This led to a significant reduction of Rab6a protein based on western blot analysis (Fig. 2A). To test the specificity of siRNA, the expression of a panel of small GTPases related to oocyte meiosis was evaluated by qRT-PCR in control and Rab6a-siRNA oocytes. As shown in Fig. 2B, the abundance of Rab6a mRNA was specifically decreased, while other gene products were not directly affected. We then analyzed how the Rab6a knockdown (Rab6a-KD) affects oocyte maturation.

During maturation, oocytes experience germinal vesicle breakdown (GVBD), and then microtubules organize into the meiosis I (MI) spindle, with chromosomes aligning at the equator plate. Following the MI division, oocytes extrude the first polar body (Pb1), and then proceed to arrest at metaphase II (MII) waiting for fertilization. Our results showed that Rab6a knockdown did not affect meiotic resumption significantly, indicated by the rate of GVBD (75.0 ± 6.4% vs. 86.7 ± 3.5% control, p > 0.05; Fig. 2B). However, the proportion of Pb1 extrusion was dramatically decreased in Rab6a-KD oocytes when compared to controls (38.2 ± 6.1% vs. 85.1 ± 3.3% control, p < 0.05; Fig. 2C). These data indicate that Rab6a is involved in the control of meiotic progression.

### Differing effects on the dynamics of Golgi apparatus and endoplasmic reticulum in Rab6a-depleted oocytes

**Figure 3:**
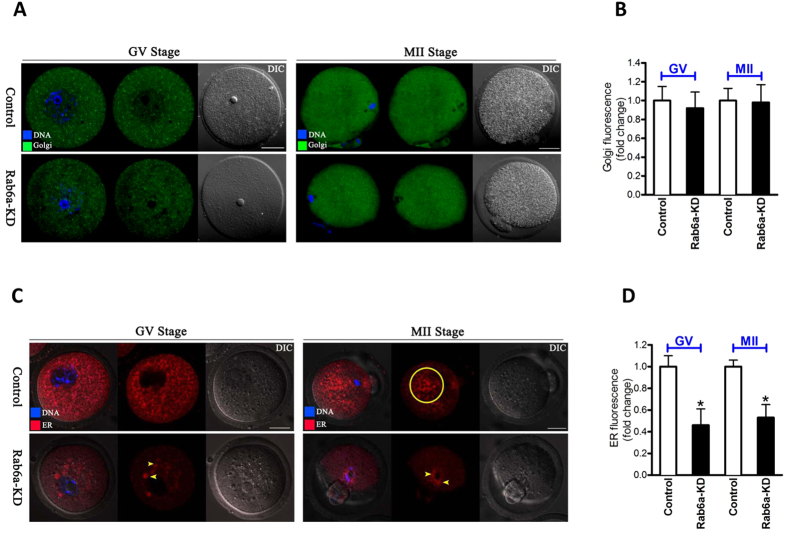
Effects of Rab6a knockdown on Golgi apparatus and endoplasmic reticulum in oocytes. Control and Rab6a-KD oocytes at GV and MII stages were labeled with GM130 antibody (green), and counterstained with Hoechst 33342 to visualize DNA (blue). (**A**) Representative confocal images showing the similar Golgi distribution pattern between control and Rab6a-KD oocytes. (**B**) Quantification of Golgi fluorescence from control and Rab6a-KD oocytes. (**C**) Representative confocal images showing the altered ER distribution (red) in Rab6a-KD oocytes compared to controls. Circle indicates the polarized ER in control oocytes. Arrowheads indicate the clustered ER in Rab6a-KD oocytes. (**D**) Quantification of ER fluorescence from control and Rab6a-KD oocytes. Data are expressed as the mean ± SD from three independent experiments in which at least 50 oocytes were analyzed. Scale bar, 25 μm. *p < 0.05 vs controls.

Since it is known that Rab proteins are the link between Golgi organization and membrane trafficking in humans and yeast^18^, we decided to examine the relationship between Rab6a and Golgi dynamics in mouse oocytes. By staining with GM130 antibody, we found the apparent and uniform distribution of Golgi in cytoplasm of both GV and MII oocytes, as shown in Fig. 3A. Surprisingly, Rab6a knockdown seemed to have little effects on the status of Golgi complex including distribution and intensity (Fig. 3A,B), indicating that Rab6a is not likely to be involved in Golgi organization in oocytes.

Dynamic redistribution of endoplasmic reticulum (ER) has been suggested to be necessary for producing a competent oocyte^19^, we therefore further examined whether ER localization was influenced in Rab6a-KD oocytes. To test this hypothesis, we stained GV and MII stage oocytes with ER tracker and imaged them by confocal microscope. In control oocytes, ER was distributed throughout the cytoplasm in a network manner at GV stage (Fig. 3C). When the oocytes proceeded to MII stage, ER presented a more polarized pattern around chromosome region (Fig. 3C, circle). Remarkably, as compared to control group, we frequently observed the ER clustering in both GV and MII oocytes with Rab6a depletion (arrowheads; Fig. 3C). Furthermore, quantitative analysis of fluorescence intensity also showed that ER signals reduced by 50% when Rab6a was knocked down (Fig. 3D). Together, these results suggest that Rab6a is required for ER reorganization during mouse oocyte maturation.

### Rab6a depletion disrupts the formation of cortical granule free domain during oocyte maturation

**Figure 4:**
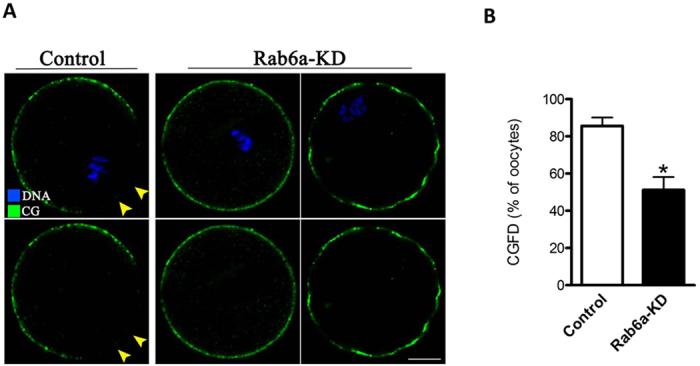
Rab6a knockdown disrupts the formation of cortical granule free domain in oocytes. MII oocytes were labeled with FITC-conjugated lectin to visualize cortical granules (green) and counterstained with Hoechst 33342 for chromosomes (blue). (**A**) Representative confocal images showing the distribution of CGs in control and Rab6a-KD oocytes. Arrowheads indicate the presence of cortical granules free domain (CGFD). (**B**) Quantitative analysis of control and Rab6a-KD oocytes with CGFD. At least 30 oocytes were examined for each group, and data are expressed as the mean ± SD from three independent experiments. Scale bar, 25 μm. *p < 0.05 vs controls.

Cortical granules (CGs) are membrane bound organelles and are rich in proteinases and carbohydrates. CG redistribution in oocytes has been used as an important criterion to evaluate cytoplasmic maturation^20^. As the resumption of meiosis, the CGs undergo significant changes in their cortical location, leading to the formation of a CG-free domain (CGFD) around the metaphase spindle^21^. To evaluate the effects of Rab6a on CG dynamics, GVBD +7 hours oocytes were labeled with CG marker and examined by confocal microscope. As shown in Fig. 4, about 85% of control oocytes displayed a typical CGFD, indicated by arrowheads (Fig. 4A). In striking contrast, we observed the CGFD in only around 50% of Rab6a-KD oocytes, which is significantly lower than controls (Fig. 4B). Two abnormal distribution patterns of CGs were detected in Rab6a-KD oocytes, including intact distribution and discontinuous distribution, as shown in Fig. 4A. GVBD +10 hours (MII) oocytes were also examined for the CGFD formation, and similar phenotypes were observed (data not shown). These results implicated that Rab6a depletion disrupts the formation of CGFD during mouse oocyte maturation.

### Intracellular Ca^2+^ stores are decreased in Rab6a-depleted oocytes

**Figure 5:**
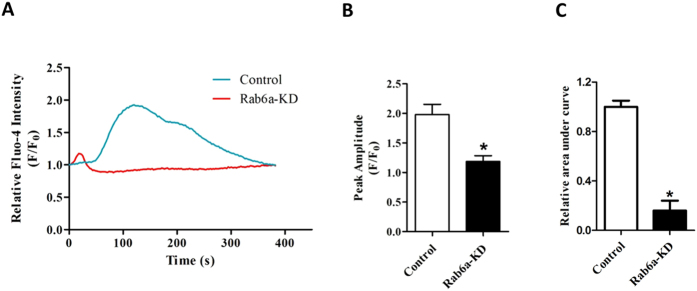
Rab6a-depleted oocytes demonstrate a decreased Ca^2+^release compared to controls. Control and Rab6a-KD oocytes at MII stage were loaded with Fluo-4 AM, treated with ionomycin and imaged by confocal microscope. (**A**) Representative curve showing the ionomycin-induced Ca2+ release. (**B,C**) Histograms showing the maximum amplitude and area under the curve in Rab6a-KD and control oocytes. At least 25 oocytes were examined for each group, and data are expressed as the mean ± SD from three independent experiments. *p < 0.05 vs controls.

It is well known that ER is important for Ca^2+^ signaling at fertilization. The finding that Rab6a-KD oocytes contain the altered distribution pattern of ER, led to the hypothesis that ER function, particularly as a Ca^2+^ source, might be compromised accordingly in Rab6a-KD oocytes. Hence, we measured Ca^2+^ release from intracellular stores in oocytes to determine if Ca^2+^ storage was changed when Rab6a was knocked down. For this purpose, MII stage oocytes were labeled with Fluo-4, a Ca^2+^ sensitive dye, and treated with ionomycin to release Ca^2+^ from stores. Changes in Fluo-4 intensity were normalized to the resting Ca^2+^ levels and presented as F/F0. As shown in Fig. 5A, we found that control oocytes treated with ionomycin resulted in an ~2 fold increase in Fluo4 intensity, while no increased signal was detected in Rab6a-KD oocytes responded to ionomycin. Both area under the curve and maximum amplitude of the Ca^2+^ release were significantly reduced in Rab6a-siRNA oocytes relative to controls (Fig. 5B,C). Collectively, these data imply that the disrupted redistribution of organelles in Rab6a-KD oocytes might contribute to the reduced calcium storage.

### Failure to form actin cap in Rab6a-depleted oocytes

**Figure 6:**
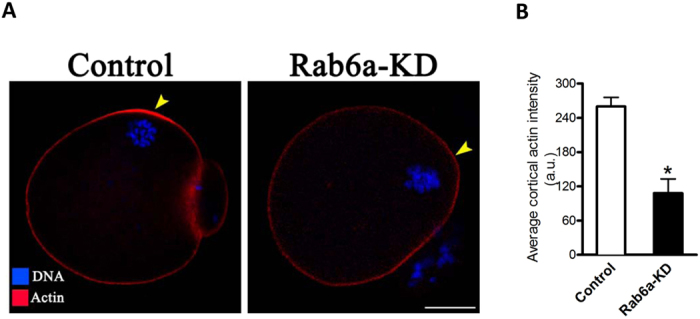
Formation of actin cap requires Rab6a during oocyte meiosis. MII oocytes were labeled with phalloidin to visualize actin (red) and counterstained with Hoechst 33342 for chromosomes (blue). (**A**) Representative images showing the actin distribution in control and Rab6a-KD oocytes. Arrowheads indicate the position of actin cap. (**B**) Quantification of cortical actin intensity of control and Rab6a-KD oocytes. At least 50 oocytes were examined for each group, and data are expressed as the mean ± SD from three independent experiments. Scale bar, 25 μm. *p < 0.05 vs controls.

Dynamic microfilament polymerization is essential for various stages of mammalian oocyte maturation, including asymmetric spindle migration, cortical actin cap formation, and cytokinesis^22^. The formation of cortical actin cap is one of the predominant features of oocyte polarization. To investigate the effects of Rab6a knockdown on actin polymerization, matured oocytes were loaded with phalloidin and counterstained for chromosomes, and then quantitative analysis was conducted. As shown in Fig. 6A, in normal MII oocytes, actin caps are clearly formed on the membrane approaching to chromosomes (arrowhead). By contrast, actin caps were almost disappeared in Rab6a-KD oocytes, as evidenced by the markedly reduced intensity of phalloidin signals (arrowhead; Fig. 6A,B). These data suggest that Rab6a is critical for the filament polymerization in oocyte meiosis.

### Proper microtubule organization in mouse oocyte requires Rab6a

**Figure 7:**
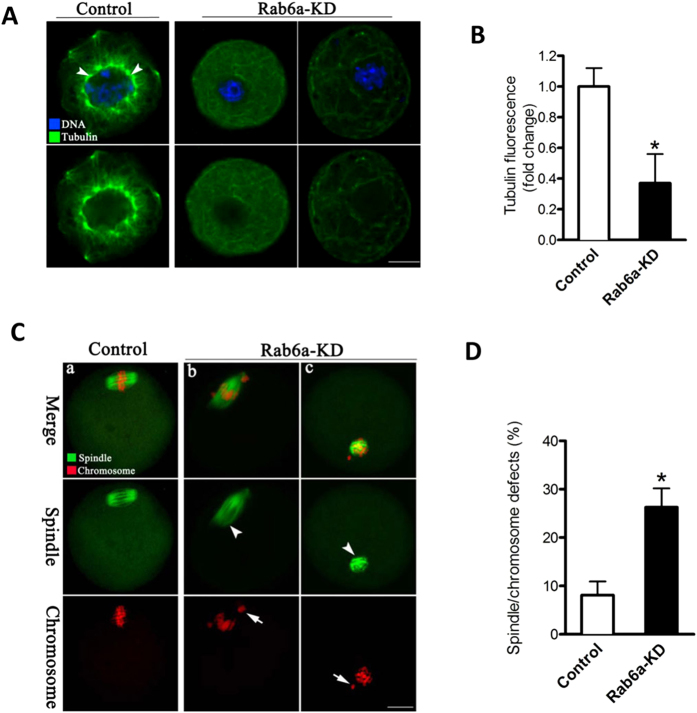
Rab6a knockdown results in the microtubule disorganization in oocytes. (**A**) Oocytes at GV stage were immunostained with α-tubulin antibody (green) and counterstained with Hoechst 33342 (blue). Representative confocal images showing the organization of microtubules in control and Rab6a-KD oocytes. Arrowheads indicate the strong signals of tubulin around germinal vesicle. (**B**) Quantitative analysis of tubulin fluorescence in control and Rab6a-KD oocytes. (**C**) Oocytes at MII stage were immunostained with α-tubulin antibody to visualize spindle (green) and counterstained with propidium iodide for chromosomes (red). Representative confocal images showing the spindle morphology and chromosome alignment in control (a) and Rab6a-KD (b-c) oocytes. Arrowheads indicate the abnormal spindle and arrows indicate the misaligned chromosomes. (**D**) Quantification of control and Rab6a-KD oocytes with spindle/chromosome defects. Data are expressed as mean percentage ± SD from three independent experiments in which at least 100 oocytes were analyzed. Scale bar, 25 μm. *p < 0.05 vs controls.

Given that microtubules are the major drivers of organelle redistribution during oocyte maturation, we decided to test whether Rab6a knockdown effects the microtubule dynamics in mouse oocytes. To do this, immature oocytes were pretreated with 0.1% Triton X-100 and then immunolabeled with anti-tubulin antibody. Confocal imaging revealed that microtubules distributed mainly around germinal vesicle, and also presented a microtubule network in the cytoplasm of control oocytes (Fig. 7A, arrowheads). It is worth noting that, when Rab6a was abated, such an organization pattern of microtubules in oocytes was evidently impaired, as shown in Fig. 7A. Meantime, we noticed that the amount of Triton-insoluble tubulin was decreased in Rab6a-KD oocytes based on the quantitative analysis of fluorescence intensity (Fig. 7B).

Next, we further assessed the effects of Rab6a knockdown on microtubule status in metaphase cells. To this end, MII oocytes were immunostained with anti-tubulin antibody to visualize the spindle and co-stained with propidium iodide to visualize chromosomes. Confocal microscopy showed that most control oocytes presented a typical barrel-shape spindle and well-aligned chromosomes at the equator (Fig. 7Ca), while Rab6a-KD oocytes displayed an increased proportion of chromosome misalignment and spindle morphology defects (26.3 ± 3.9% vs. 8.1 ± 2.8% control, p < 0.05; Fig. 7Cb,c, indicated by arrows and arrowheads, respectively). Altogether, these findings revealed that Rab6a plays an important role in microtubule stability and organization during mouse oocyte maturation.

## Discussion

Members of the large family of Rab GTPases have been demonstrated to be critical for defining the identity and transport routes of vesicles and organelles. Different Rab GTPases recruit different effector proteins to guide vesicles to their correct destination^23^,^24^. Among the 60 or more members of Rab family in mammalian cells, Rab6 is one of the most extensively studied Rab proteins involved in regulating Golgi trafficking, maintaining its integrity, and its steady-state homeostasis^18^. Unexpectedly, in this study, we found that Rab6a knockdown in mouse oocytes did not show obvious effects on the distribution and intensity of Golgi apparatus (Fig. 3). Rab33b, Rab18, and Rab43 have been reported to have a role in the regulation of Golgi trafficking and organization. For example, overexpression of RN-tre, which is the GAP for Rab43, leads to the inhibition of Shiga toxin trafficking from cell surface to trans-Golgi and Golgi ribbon fragmentation^25^. Rab18 knockdown causes inhibition of transport of the model cargo to the cell surface and visible Golgi fragmentation^26^. Based on these findings, it is possible that other potential Rab proteins, not Rab6a, is the major regulator of Golgi apparatus in mouse oocytes.

In striking contrast, we observed the defective organization and decreased abundance of ER in oocytes depleted of Rab6a (Fig. 3C,D). This finding is consistent with the previous results derived from other cell types^27^. During maturation, mouse oocytes experience a ER rearrangement from a more evenly distributed pattern at GV stage to a more polarized pattern at MII stage^28^. Cytoskeleton has recently emerged as a critical regulator ER dynamics and its network remodeling. Although ER can form a reticular network independently of cytoskeletal structures, ER distribution and sheet/tubule balance are influenced by microtubules in mammalian cells^29^. There also is a growing number of reports indicating that actin plays a significant role in ER structure in somatic cells, for example: ER has been shown to align along actin fibers in kidney epithelial cells^30^, and interact with actin filaments in insect photoreceptor cells^31^. Fitzharris *et al.,* revealed that ER reorganization in mouse oocytes is a complex multi-step process involving distinct microtubule- and microfilament-dependent phases^28^. Notably, here our data showed that Rab6a knockdown markedly disrupts the formation of actin cap and assembly of microtubule network during oocyte maturation (Figs 6 and 7). Likewise, Rab6a was demonstrated to regulate the microtubule-dependent recycling at the trans-Golgi network^32^, and to inactivate spindle assembly checkpoint (SAC) in mitosis^16^. Taken together, these findings support a model where Rab6a, likely through interaction with cytoskeletal structures, promotes the proper organization of organelles and vesicles in mammalian oocytes.

Following sperm penetration, cortical granules (CGs), a kind of special organelle in ovulated eggs, released their contents into the perivitelline space in an event that is termed the cortical reaction, which is a critical step to block polyspermic penetration^33^. In this study, we observed that this unique distribution pattern of CGs in oocytes was altered when Rab6a was abated (Fig. 4). In *C. elegans*, Rab6 recruits separase to CG for exocytosis during oocyte-to-embryo transition^17^. Also noteworthy is that, Rabphilin-3A, a putative target protein for Rab3A, has been shown to participate in Ca^2+^-dependent CG exocytosis in mouse eggs^34^,^35^. At fertilization, the release of intracellular Ca^2+^ is necessary and sufficient for most, if not all, of the major events of egg activation. One of the earliest events that is induced by Ca^2+^ rise is CG exocytosis^36^. Importantly, Rab6a knockdown resulted in a remarkable decrease in Ca^2+^ stores in oocytes (Fig. 5), which is perhaps associated with the impaired ER function. In turn, such a lowered Ca^2+^ content in oocytes would inevitably affects the following fertilization as well as embryonic development.

The mammalian oocyte undergoes two consecutive rounds of extremely asymmetric divisions to generate a haploid egg. Such divisions are dictated by intracellular asymmetries developed within the oocyte, including spindle positioning, actin cap and cortical polarization^37^. To promote spindle and chromosome positioning, the cortical actin polymerization not only drives cytoplasmic streaming, but also suppresses premature contraction of the myosin II ring around the actin cap^38^. Errors in chromosome segregation in oocytes lead to embryo aneuploidy, which contributes to early pregnancy loss. At the heart of chromosome segregation is the spindle, a dynamic biomechanical machine fashioned from microtubules^39^. In the present study, we showed the spindle defects, chromosome misalignment and actin cap loss in Rab6a-KD oocytes (Figs 6 and 7), suggesting that Rab6a is a novel regulator of these meiotic structures. Nonetheless, the underlying mechanisms on how Rab6a modulates meiosis in mammalian oocytes remain to be explored.

## Materials and Methods

All chemicals and culture media were purchased from Sigma (St. Louis, MO, USA) unless stated otherwise. ICR mice were used in this study. All experiments were approved by the Animal Care and Use Committee of Nanjing Medical University and were performed in accordance with institutional guidelines.

### Antibodies

Mouse monoclonal anti-Rab6a (Cat# ab55660) and GM130 antibody (Cat# ab52649) were purchased from Abcam (Cambridge, MA, USA); Mouse monoclonal anti-α-tubulin-FITC antibody (Cat# 76074), Phalloidin-FITC (Cat# P5282) and Lectin-FITC (Cat# L4265) were purchased from Sigma (St. Louis, MO, USA); FITC-conjugated goat anti-rabbit IgG was purchased from Thermo Fisher Scientific (Rockford, IL, USA). ER-tracker red (Cat# C1041-1) was purchased from Beyotime, Fluo-4 AM (Cat# F14201), and Pluronic F-127 (Cat# P-3000MP) were purchased from Thermo Fisher Scientific.

### Oocyte collection and culture

Approximately 46–48 h after injection of 5 IU Pregnant Mares Serum Gonadotropin (PMSG), fully-grown immature oocytes were harvested from ovaries of 6-8 week old female ICR mice. Surrounding cumulus cells were removed by repeatedly pipetting, and then oocytes were cultured in M16 medium under mineral oil at 37 °C in a 5% CO_2_ incubator. AT appropriate time points, oocytes were selected for the following assays.

### siRNA knockdown

Microinjection of siRNA into the cytoplasm of fully-grown immature oocytes was used to knock down Rab6a. siRNA (GenePharma, Shanghai, China) was diluted with water to give a stock concentration of 1 mM, and then 2.5 picoliters were injected injected into oocytes with a Narishige microinjector. A siRNA negative control was injected as control.

Rab6a-siRNA:

5′- GGAGCAACCAGUCAAUGAATT-3′;

5′-UUCAUUGACUGGUUGCUCCTT-3′

Negative control siRNA:

5′-UUCUCCGAACGUGUCACGUTT-3′;

5′-ACGUGACACGUUCGGAGAATT-3′

After injections, oocytes were arrested at GV stage with 2.5 μM milrinone for 20 hours, and then were cultured in milrinone-free M2 medium for maturation.

### Western blotting

100 oocytes were lysed in Laemmli sample buffer containing protease inhibitor, and boiled for 5 min before subjected to 10% SDS-PAGE. A PVDF membrane was used to transfer the separated proteins, then blocked in TBST (TBS containing 0.1% Tween 20) with 5% nonfat milk for 1 hour. Then the PVDF membrane was separated and incubated overnight at 4^o^C with primary antibodies as follows: rabbit anti-Rab6a antibody (1:1000) and anti-tubulin antibody (1:3000). After washes in TBST, membranes were incubated with HRP-conjugated secondary antibodies for 1 hour at room temperature, then processed using an ECL Plus Western Blotting Detection System.

### Quantitative real-time PCR

Total RNA was isolated from 100 oocytes using an RNAqueous-Micro Kit (Ambion, TX, USA), and cDNA was quantified by qRT-PCR using an ABI Stepone Plus Real-time PCR system (Applied Biosystems, CA, USA). The fold change in gene expression was calculated using the ΔΔCt method with the house keeping gene, glyceraldehydes-3-phosphate dehydrogenase (GAPDH), as the internal control. Primer sequences are listed in Supplemental Table I.

### Immunofluorescence and Confocal microscopy

For staining of Rab6a, Golgi, actin, and CGs, oocytes were fixed with 4% paraformaldehyde for 30 minutes and then permeabilized with 0.5% Triton X-100 for 20 minutes. After 1 hour blocking in 1% BSA-supplemented PBS, samples were incubated overnight at 4 °C with primary antibodies as follows: anti-Rab6a antibody, anti-GM130 antibody, FITC-conjugated anti-tubulin antibody, Lectin-FITC and Phalloidin-FITC. For microtubule staining, GV oocytes were extracted for 10 min with 0.1% Triton X-100 before fixation, and then stained with anti-tubulin antibody. After three washes in PBS, oocytes were labeled with Alexa Fluor 488 goat-anti mouse IgG at room temperature for 1 hour. Propidium Iodide (PI; red) or Hoechst 33342 (blue) was used for chromosome staining. Oocyte samples were mounted on anti-fade medium (Vectashield, Burlingame, CA, USA), and then examined under a Laser Scanning Confocal Microscope (LSM 710, Zeiss, Germany) equipped with the 40x objectives.

For staining of endoplasmic reticulum, oocytes were loaded with ER-tracker red (1:1000) in HEPES-buffered Tyrode’s solution (HBTS; 119 mM NaCl, 5 mM KCl, 25 mM HEPES buffer, 2 mM CaCl_2_, 2 mM MgCl_2_, 6 g/L glucose, 0.1 g/L PVA, and adjust pH to 7.4 with NaOH) with Hoechst 33342 for 30 minutes at 37 °C. Oocytes were then washed with HBTS only and attached to Mattek dish for imaging under a LSM. Image J software (NIH) was used to quantify fluorescence intensity as previously described^8^. Fluorescence intensity was randomly measured in at least five regions of interest strictly limited to the cytoplasm (for ER and tubulin) or cortex (for actin) of each oocyte images. Fluorescence signal was calculated as the average intensity after background subtraction.

### Ca^2+^ imaging

For Ca^2+^ imaging, oocytes were loaded with 1.5 μM Fluo-4 AM and 0.02% Pluronic F-127 in Ca^2+^ free HEPES-buffered Tyrode’s solution (HBTS) containing 1 mM EGTA for 20 min at 37 °C. Following two washes with HBTS, oocytes were attached to Mattek dish for imaging. After baseline signals were recorded, ionomycin (1 μM) was added to release Ca^2+^ from stores. Once ionomycin was added, images were taken every 2.5 second immediately, using a Laser Scanning Confocal Microscope (LSM 710, Zeiss, Germany) equipped with the 40x objectives. Changes in Fluo-4 intensity were normalized to resting Ca^2+^ levels and presented as F/F0. Ca^2+^ imaging were recorded from at least 25 oocytes each group in 3 independent experiments.

### Statistical analysis

Data are presented as mean ± SD, unless otherwise indicated. Statistical comparisons were made with Student’s *t* test and ANOVA when appropriate. P < 0.05 was considered to be significant.

## Additional Information

**How to cite this article**: Ma, R. *et al*. Involvement of Rab6a in organelle rearrangement and cytoskeletal organization during mouse oocyte maturation. *Sci. Rep.* **6**, 23560; doi: 10.1038/srep23560 (2016).

## Supplementary Material

Supplementary Information
