# Supplementary material for: Involvement of Rab6a in organelle rearrangement and cytoskeletal organization during mouse oocyte maturation
Source: Sci Rep. 2016 Mar 31;6:23560. doi: 10.1038/srep23560 (PMC4814827; doi:10.1038/srep23560)
Supplement: Supplementary Information [file srep23560-s1.doc]

**Involvement of e ns of Rab5a-positive vesicles000000000000000000000000000000000000000000000000000000000000000000000000000000000000000000000000Rab6a in organelle rearrangement and cytoskeletal organization during mouse oocyte maturation**

Rujun Ma^1,2,3^, Jiaqi Zhang^2^, Xiaohui Liu^1,2^, Ling Li^2^, Honglin Liu^1^, Rong Rui^4^, Ling Gu^1,*^, Qiang Wang^2,*^

^1^College of Animal Science & Technology, Nanjing Agricultural University, Nanjing, China; ^2^State Key Laboratory of Reproductive Medicine, Nanjing Medical University, Nanjing, China; ^3^Center of Reproductive Medicine, Jinling Hospital, Medical School of Nanjing University, Nanjing, China; ^4^College of Veterinary Medicine, Nanjing Agricultural University, Nanjing, China.

***Correspondence should be addressed to:**

Qiang Wang, Ph.D., Professor

State Key Laboratory of Reproductive Medicine

Nanjing Medical University

140 Hanzhong Rd, Nanjing, Jiangsu, 210029 China

Phone: +86-25-86862151; Fax: +86-25-86862151

E-mail: qwang2012@njmu.edu.cn

**OR**

Ling Gu, Ph.D., Associate Professor

College of Animal Science & Technology

Nanjing Agricultural University

1 Weigang, Nanjing, Jiangsu, 210095 China

Phone: +86-25-84399690

E-mail: lgu@njau.edu.cn

# Supplemental Table 1 Primer sequences of genes for qRT-PCR

***Gene Primer sequence***

GAPDH Forward Primer: 5’ –CTTTGTCAAGCTCATTTCCTGG – 3’

Reverse Primer: 5’ –TCTTGCTCAGTGTCCTTGC – 3’

Rab5a Forward Primer: 5’ –TGGTCAAGAACGGTATCATAGC – 3’

Reverse Primer: 5’ –GCCTTTGAAGTTCTTTAACCCAG – 3’

Rab6a Forward Primer: 5’ –CTTCCCTGTTCTTCCCTTTCTC – 3’

Reverse Primer: 5’ –TAGCCTGGAGCTGTCTGTAA – 3’

Rab11 Forward Primer: 5’ –GTGGGCAATAAGAGTGATTTACG– 3’

Reverse Primer: 5’ –TCTGTTAGAATTGTCTGAAAAGCAG– 3’

CDC42 Forward Primer: 5’ –CATGTCTCCTGATATCCTACACAAC– 3’

Reverse Primer: 5’ –TGTCATAATCCTCTTGCCCTG – 3’

Rab3a Forward Primer: 5’ –TGGGCTTCATCCTAATGTATGAC– 3’

Reverse Primer: 5’ –TCGCTCATCTTCCATGTCAC – 3’
